# Long COVID endotheliopathy: hypothesized mechanisms and potential therapeutic approaches

**DOI:** 10.1172/JCI161167

**Published:** 2022-08-01

**Authors:** Jasimuddin Ahamed, Jeffrey Laurence

**Affiliations:** 1 Cardiovascular Biology Research Program, Oklahoma Medical Research Foundation, Oklahoma City, Oklahoma, USA.; 2 Department of Medicine, Division of Hematology and Medical Oncology, Weill Cornell Medicine, New York, New York, USA.

## Abstract

SARS-CoV-2–infected individuals may suffer a multi–organ system disorder known as “long COVID” or post-acute sequelae of SARS-CoV-2 infection (PASC). There are no standard treatments, the pathophysiology is unknown, and incidence varies by clinical phenotype. Acute COVID-19 correlates with biomarkers of systemic inflammation, hypercoagulability, and comorbidities that are less prominent in PASC. Macrovessel thrombosis, a hallmark of acute COVID-19, is less frequent in PASC. Female sex at birth is associated with reduced risk for acute COVID-19 progression, but with increased risk of PASC. Persistent microvascular endotheliopathy associated with cryptic SARS-CoV-2 tissue reservoirs has been implicated in PASC pathology. Autoantibodies, localized inflammation, and reactivation of latent pathogens may also be involved, potentially leading to microvascular thrombosis, as documented in multiple PASC tissues. Diagnostic assays illuminating possible therapeutic targets are discussed.

## Introduction

Reported COVID-19 deaths between January 1, 2020, and December 31, 2021, totaled 5.94 million worldwide, but that is a vast underestimation (1). As measured by excess mortality, it is likely that 18.2 million died worldwide because of COVID-19 over that period (1). A substantial portion of individuals infected with SARS-CoV-2, the etiologic agent of COVID-19, also suffer multi-organ dysfunction following recovery from acute COVID-19 (2, 3). The term “long COVID” was coined in May 2020 by patients to refer to post-acute conditions (4, 5). It may be the first illness with specific symptom clusters recognized by patients after finding one another on Twitter (5). Also known as post-acute sequelae of SARS-CoV-2 infection (PASC), it was first studied among severe COVID-19 patients, approximately 40% of whom were unable to return to normal activities 60 days after hospital discharge (6). But PASC also complicates recovery from mild or asymptomatic COVID-19 (7–9). Although most of the PASC cases reviewed here derive from infection with the original SARS-CoV-2 strain or its earliest variants, there is no evidence that recent isolates are less frequent harbingers of PASC. Indeed, one medical editorial admonished that “current numbers and trends indicate that ‘long-haul Covid’ (or ‘long Covid’) is our next public health disaster in the making” (10). The pathophysiology is unknown. Multiple lines of evidence implicate endotheliopathy in association with cryptic SARS-CoV-2 tissue reservoirs in PASC, but the mechanism for this endothelial cell (EC) injury is uncertain. It may involve SARS-CoV-2 RNA fragments and soluble proteins, specific autoantibodies, local proinflammatory cytokine signaling, and reactivation of latent pathogens. In addition, such injury may lead to a microvascular thrombosis underlying organ dysfunction, for which there is accumulating evidence in diverse PASC tissues.

There is no internationally recognized case definition for PASC, and this complicates pathophysiologic investigations. The CDC requires only an interval of more than 4 weeks after acute COVID-19 (11), on the basis of which one in five COVID-19 survivors aged 18–64 years, and one in four older than 65 years, experienced an incident PASC condition (11, 12). Using a prospective Delphi consensus-seeking exercise and a mixed iterative survey of experts and patients, the WHO developed a definition requiring a longer interval: “Post COVID-19 condition occurs in individuals with a history of probable or confirmed SARS-CoV-2 infection, usually 3 months from the onset of COVID-19 with symptoms that last for at least 2 months and cannot be explained by an alternative diagnosis.... Symptoms may be new onset, following initial recovery from an acute COVID-19 episode, or persist from the initial illness. Symptoms may also fluctuate or relapse over time” (13).

**Figure 1:**
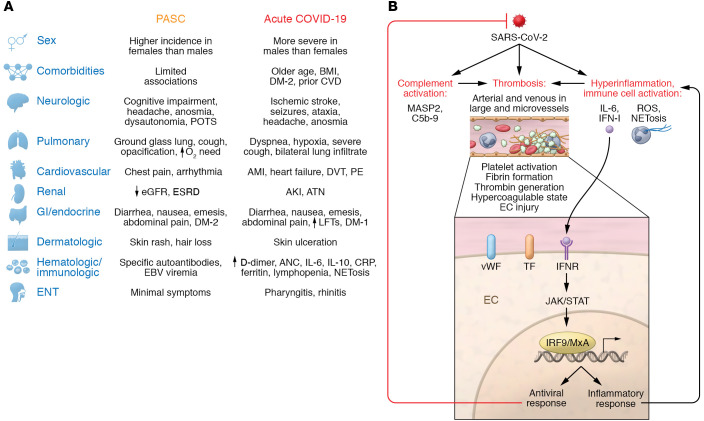
Clinical signs and symptoms distinguish long COVID (PASC) from acute COVID-19. (**A**) Common clinical signs and symptoms as well as comorbidities and other cofactors for disease progression distinguish long COVID, also known as PASC, from acute COVID-19. In particular, female sex at birth is linked to a higher incidence of PASC, while male sex at birth is a risk factor for acute COVID-19 progression. Multiple metabolic and cardiovascular risk factors exacerbate acute COVID-19. Overall, age, BMI, and prior respiratory or cardiovascular history do not affect the incidence of PASC but may influence its clinical phenotype. (**B**) An acute thromboinflammatory process characterizes acute COVID-19. Clinical progression parallels biomarkers of EC injury and a hyperinflammatory state, including enhanced release of proinflammatory cytokines and chemokines, activation of complement and coagulation cascades, platelet activation, NETosis, and, ultimately, hypoxia. IFN-I signals can promote an antiviral response via MxA and exacerbate inflammation. AKI, acute kidney injury; AMI, acute myocardial infarction; ANC, absolute neutrophil count; ATN, acute tubular necrosis; CRP, C-reactive protein; CVD, cardiovascular disease; DM-1, DM-2, diabetes mellitus types 1 and 2; DVT, deep vein thrombosis; eGFR, estimated glomerular filtration rate; ENT, ear, nose, and throat; ESRD, end-stage renal disease; LFTs, liver function tests; NET, neutrophil extracellular trap; PASC, post-acute sequelae of SARS-CoV-2 infection; PE, pulmonary embolism; POTS, postural orthostatic tachycardia syndrome; TF, tissue factor; vWF, von Willebrand factor.

Divergent PASC phenotypes also complicate investigation of PASC epidemiology and pathophysiology (11, 12, 14). Recent surveys of large population groups post–acute COVID-19 that include pre-pandemic comparator groups and involvement of more than one organ system suggest a PASC incidence of approximately 14% in adults (15) and approximately 21% in children and adolescents (16). In contrast, studies reporting much higher incidence rates often lack objective findings documented by quantitative assays (16, 17). In exploring hypotheses in this Review, we focus on those PASC features for which objective, quantitative measures are available (Figure 1).

## Formulating hypotheses for PASC pathogenesis

### Clinical and immunologic manifestations of COVID-19 versus PASC.

Based on some similarities to acute COVID-19, initial speculation on PASC pathophysiology focused on systemic inflammation. Acute COVID-19 severity parallels biomarkers of EC injury and a hyperinflammatory state, including enhanced release of proinflammatory cytokines and chemokines, activation of complement and coagulation cascades, platelet activation, and neutrophil extracellular trap formation (NETosis), leading to tissue hypoxia (2, 18, 19) (Figure 1). Our group was among the first to document a pattern of small-vessel arterial and venous thrombi, in association with striking deposition of complement components C5b-9, C4d, and MASP2, and tissue damage consistent with complement-mediated microvascular injury (20). This was noted both at autopsy, in the lung, and premortem, in the normal-appearing skin of individuals with severe COVID-19 (20). These data are consistent with the facts that arteriolar thrombi are more prevalent in lungs (21) and skin (22) of severe/critical COVID-19 patients versus lungs of those with other forms of acute respiratory distress syndrome (ARDS) and that microthrombi are five-fold more prevalent in hearts of patients dying of COVID-19 versus controls, with or without preexisting cardiac disease (23).

Along with autopsy evaluation of multiple organ systems (24, 25), these findings support the hypothesis that systemic venous and arterial microthrombi represent a unique characteristic of COVID-19 ARDS versus other severe respiratory infections and are a prominent cause of death (25). Anticoagulants have been used in sepsis-associated ARDS to reduce macrovessel thromboembolism, but most randomized trials to date have not shown benefit of add-on or escalated antithrombotic therapy over usual standard of care in critically ill acute COVID-19 patients (26, 27). There is a survival benefit with therapeutic-dose heparin in the noncritically ill COVID-19 patient (28). However, hemostatic and thromboinflammatory biomarkers are similar in hospitalized COVID-19 patients with or without use of prophylactic low–molecular weight heparin (29). Classic antithrombotics reduce macrovessel thrombosis but may not be effective in ameliorating the microvessel thrombosis in SARS-CoV-2 infection (26).

Elevated levels of proinflammatory cytokines, chemokines, and IFN-I–related products correlate with vasculopathy and adverse clinical outcomes in acute COVID-19 (30, 31). These factors could initiate and perpetuate thromboinflammatory cascades, leading to EC activation and injury, leukocyte recruitment, thrombin generation, platelet activation, and fibrin formation, causing thrombosis (Figure 1B). Gastrointestinal (GI) dysfunction, manifest by diarrhea and common in both acute COVID-19 (32) and PASC (2, 3, 8), may facilitate this pathology via perpetuation of a proinflammatory state triggered by microbial translocation secondary to compromise of the GI mucosal barrier by SARS-CoV-2 infection of epithelial lining cells (32). This process has been linked to a multisystem inflammatory syndrome in acute COVID-19 among children (MIS-C) (33). Microbial translocation is also implicated in the persistent immune activation, despite absence of detectable viral replication, of HIV-infected individuals on antiretroviral therapy (34).

Although suppression of these inflammatory signals using dexamethasone (35), IL-1 and IL-6 inhibitors (30), anticomplement agents (36, 37), and JAK1/2 inhibitors such as baricitinib (38) had varying levels of success in mitigating the morbidity and mortality of acute COVID-19, there are no data concerning their efficacy in preventing or treating PASC. The need for new pathophysiology-based therapeutic strategies in PASC is illustrated by comparative clinical and pathologic studies described here.

### Acute COVID-19 versus PASC: hematologic and immunologic markers.

Lymphopenia, either isolated or in conjunction with an elevated absolute neutrophil count (ANC), increased lactate dehydrogenase, liver function tests, and troponins, and high levels of C-reactive protein (CRP), IL-6, IL-2 receptor, D-dimer, and ferritin are common following a symptomatic acute COVID-19 infection (39, 40). These abnormalities correlate with increased risk for clinical progression, including large-vessel deep vein thrombosis (DVT), acute myocardial infarction (AMI), and acute ischemic stroke (41) (Figure 1). In contrast, DVT, pulmonary embolism, and related venous thromboembolic disorders are not prominent features of PASC (Figure 1A), at least in individuals more than 3 months post–acute COVID-19 (42). This is discussed in the “EC pathology: PASC versus other severe respiratory infections” section below. AMI and stroke are also uncommon cardiovascular manifestations of PASC (43). In parallel, a meta-analysis of 15 publications describing 47,910 adults with long COVID found scant evidence for systemic inflammation in PASC (44). Only 3% had elevated IL-6, 8% had elevated CRP, and 8% had elevated ferritin. In two PASC cohorts, CRP and IL-6 did persist at high levels after recovery from severe COVID-19, but this was not seen following mild COVID-19 (9, 45), despite equivalent risk for PASC, at least based on some phenotypes, in the latter. A third study found that ANC, CRP, IL-6, and IL-2 receptor levels had normalized in most PASC patients, despite persistent symptoms (46). A fourth study reported only a trend toward higher IL-6 levels in PASC, and TNF-α was minimally elevated (47).

Certain PASC phenotypes may be linked to elevated systemic markers of inflammation, but the data are incomplete. Two investigations found a correlation between long COVID neurologic manifestations and increased levels of CRP and IL-6 (45), or elevated IL-4 but not IL-6 (48). In one report, IL-6 correlated with cerebral blood flow and white matter microstructure changes, but this association was found only at the *P* < 0.05 level (45). IL-6 was also not elevated in those with respiratory PASC symptoms, including CT abnormalities linked to fibrosis, although plasma TGF-β and complement membrane attack complex C5b-9 were elevated (49). The latter does characterize the vasculopathy of acute COVID-19 (20, 22).

Hematologic findings prominent in PASC include IFN-I autoantibodies, and antinuclear antibodies (ANA) against Ro, La, U1-snRNP, Jo-1, and P1 (Figure 1), the latter commonly associated with active systemic lupus erythematosus (50). The significance of the anti–nuclear antigen antibodies is unclear. One group similarly found elevated ANA titers (>1:160) in 4.3% of individuals with PASC symptoms 12 months after acute disease, but this is comparable to an approximately 5% prevalence in the general population (51).

Apart from AMI and ischemic stroke, other clinical signs and symptoms of acute COVID-19 are also distinct from PASC. Figure 1A depicts the most prevalent clinical signs, symptoms, comorbidities, and cofactors of acute COVID-19 (39, 52, 53) versus PASC (2, 3, 7, 32, 40, 42, 54–56), categorized by organ system and discussed in detail in the *Clues from studies of EC injury* section below.

### Demographic distinctions between PASC and acute COVID-19.

Comorbidities leading to increased morbidity and mortality in acute COVID-19 include advanced age, obesity, diabetes mellitus (DM), malignancy, hypertension, and atrial fibrillation (52, 53). Race/ethnicity has a complex association with acute COVID-19 and PASC, as minority populations are overrepresented in occupations at high risk for SARS-CoV-2 infection, less likely to have access to testing and health care, and less likely to be hospitalized for any given level of disease severity (4, 57). But apart from structural factors potentially impacting both disorders, the majority of epidemiologic and cohort studies find no impact of age, type 2 DM (DM-2), BMI, or hypertension on risk for PASC development in adults (11, 15, 58–60) or children (16) (Figure 1A). However, these factors must be considered in the context of acute COVID-19 severity, PASC phenotypes, ascertainment bias, and illness trajectory in hospitalized versus community-based patients, and in comparison with contemporary controls (61). Overall PASC risk may indeed be independent of initial disease severity. In contrast, multisystem illness severity in acute COVID-19, rather than preexisting comorbidities, appears to be the preeminent factor driving PASC characterized by quantifiable cardiac, pulmonary, or renal abnormalities (61).

Sexual phenotype is another principal demographic distinction between PASC and acute COVID-19. Female sex at birth offers the greatest reduction in relative risk for acute COVID-19 progression, and this holds true for adults at any age interval, through >90 years of age (53). This makes pathophysiologic sense in acute COVID-19, as male sex at birth is linked to elevated basal levels of those proinflammatory cytokines (IL-6, CRP), coagulation factors, and complement components implicated in the hypercoagulable and proinflammatory state of acute SARS-CoV-2 infection (63–65) (Figure 1B). But in PASC, the sexual-phenotype association is reversed. Two large studies examining PASC incidence in the United Kingdom (9) and China (66) found similar results: significantly higher percentages among women. Compared with men, women had odds ratios between 1.47 and 2.00 for more subjective symptoms of PASC such as fatigue, muscle weakness, anxiety, and depression and, strikingly, an odds ratio of 2.97 for more objective injury, including lower air diffusion capacity (66). This sex distinction has been replicated in numerous smaller cohorts among diverse adult populations (11, 50, 58–60). At least three studies also found female sex to be a risk factor for PASC in children and adolescents (67).

The mechanisms of this sex difference are uncertain and may relate, in part, to distinct PASC phenotypes. For example, fatigue predominates among women with PASC and has been associated with elevated IL-6, while dyspnea is prevalent among men with PASC and did not correlate with IL-6 levels (68). Sex-related differences in the innate immune system may also be involved, including the fact that the gene encoding TLR7, which activates the IFN-I antiviral response, a key defense against SARS-CoV-2 (39, 50), is on the X chromosome, and women predominate in most autoimmune phenomena (69).

### Clues from studies of EC injury.

Evidence for a persistent endotheliopathy in PASC is summarized in Table 1 and detailed here. In a cohort of 50 PASC patients examined a median of 68 days after acute COVID-19, three-quarters of whom had required initial hospitalization, there was clear biomarker evidence of sustained endotheliopathy (46). This included elevation in convalescent COVID-19 patients versus healthy controls of von Willebrand factor (vWF) antigen, vWF propeptide, and soluble thrombomodulin. Both plasma vWF antigen and propeptide levels correlated inversely with exercise capacity (6-minute walk testing) (46). In contrast, biomarkers of inflammation, including CRP, IL-6, and NETosis, assessed by DNase activity and extracellular DNA, had normalized. These results parallel two other studies. In one, cytokines reflecting vascular injury and repair, including VCAM-1, ICAM-1, and bFGF, correlated with symptoms 3 months after acute COVID-19 (70). In another, of individuals examined 4 months after COVID-19 symptom onset, no difference was found in levels of IL-6, IL-10, IP-10, sCD14, or sCD163 — the latter two are markers of monocyte activation — in those with and without PASC (71).

CT and MRI studies of PASC patients with cardiopulmonary and neurologic symptoms, reviewed below, are not sensitive enough to discriminate between localized inflammation and microthrombosis as critical components of PASC. Tissue biopsy is required. But these techniques, in combination with functional studies, offer some important clues, as discussed below.

### Cardiopulmonary data and the microthrombus hypothesis for PASC.

In one study of 47 non-hospitalized COVID-19 patients examined 67 ± 16 days after recovery, 40% of whom had cardiopulmonary PASC symptoms, focal fluorodeoxyglucose uptake by the heart on PET, consistent with myocardial inflammation, was found in only 17% (72). Initially elevated systemic inflammatory biomarkers, including IL-6, IL-8, and CRP, resolved a mean 52 ± 17 days after baseline testing (72). But evidence is accumulating for microvascular involvement in the setting of cardiopulmonary PASC. In one study, ten adults were examined a mean 11 ± 1 months after onset of mild COVID-19. A marked decrease in exercise capacity on invasive cardiopulmonary exercise testing, despite normal hemoglobin values, pulmonary function testing, resting echocardiography, and chest CT, was found in comparison with healthy, age-matched controls: 70% ± 11% versus 131% ± 45% (*P* < 0.0001) (73). Impaired systemic oxygen extraction and abnormal ventilatory efficiency slope occurred in the absence of evidence for parenchymal lung disease on CT (73). The authors concluded that this implicates a peripheral rather than central cardiac limit underlying PASC. It could reflect mitochondrial injury, as SARS-CoV-2 appears to be capable of invading at least neural cell mitochondria (74). But it would also be anticipated in the presence of persistent or progressive microthrombi (73).

Support for the importance of microthrombosis in PASC also derives from studies of air trapping in such patients (75, 76). It is a common finding in bronchiolitis obliterans, linked to small airway inflammation and/or fibrosis with compression of pulmonary microvessels (75). It is also consistent with endothelial injury and alveolar capillary microthrombosis (76). In a parallel study, an adolescent with respiratory symptoms more than 7 months after acute COVID-19 and no evidence for structural damage on chest CT and cardiac MRI had significant perfusion defects on ventilation-perfusion single-photon emission CT (V/Q SPECT) (77). This was said to be “consistent with microemboli caused by microvascular and endothelial damage” (77).

The number of circulating endothelial colony-forming cells (ECFCs), an indicator of ongoing vascular damage, was also increased in individuals 3 months after SARS-CoV-2 infection compared with controls, regardless of whether a prior pulmonary thrombosis had occurred (78). PASC patients with lower PaO_2_ levels at admission had higher numbers of ECFCs (78). Such abnormalities are consistent with the decline in function of circulating ECFCs of men undergoing penile prosthesis placement for erectile dysfunction (ED) related to PASC but not for ED unrelated to COVID-19 (79). ED paralleled decreased endothelial oxide synthetase expression, indicative of compromised vascular integrity, in the penile microvasculature of PASC patients versus those with ED apart from SARS-CoV-2 (79). These findings are of interest in the context of recently identified circulating “microclots” or amyloid deposits (which are resistant to fibrinolysis and can block microvessels) in PASC, accompanied by increased antiplasmin levels (80).

### CNS data and the microthrombus hypothesis for PASC.

Cerebral microhemorrhages associated with hypoxic brain injury and neuronal degeneration are features of severe acute COVID-19 as well as nonhuman primate models of acute SARS-CoV-2 infection (81). Indirect evidence suggests that a similar neuropathology contributes to neurologic sequelae in PASC (81). In one study of 100 adults with mild COVID-19 and self-reported complications more than 6 weeks after recovery, including nonspecific cognitive complaints (“brain fog”) in 81%, headache (68%), and paresthesia (60%), patients performed significantly worse on attention and working memory cognitive testing (82). The authors speculated that intracerebral microthrombi contributed to these symptoms in PASC, as they had demonstrated by transcranial Doppler ultrasound in acute COVID-19 patients with similar symptoms (83). Disabling peripheral neuropathy, with axonal and demyelinating injury documented on skin biopsy and electrodiagnostic testing, has also been recognized in PASC, but evidence for microthrombosis was not sought (84). Finally, changes in gray matter morphometry, cerebral blood flow, and white matter microstructure by MRI were seen in patients with severe or mild COVID-19 (45). Hypoperfusion on arterial spin label was noted across the gray matter cortex, most prominent after severe COVID-19, suggesting microthrombosis (45). The latter is consistent with a recent report of retinal vein occlusions in PASC (85).

Examination of cerebrospinal fluid (CSF) from individuals with cognitive deficits developing 1 to 6 months after recovery from only mild COVID-19 confirmed that a prolonged inflammatory response is not a major factor in neurologic PASC (58). CSF leukocyte counts, glucose, CSF/serum albumin ratio, and IgG levels were within normal limits. However, abnormal oligoclonal banding patterns were identified in 69% of PASC patients with cognitive deficits versus none of the controls (58) or acute COVID-19 patients (86). The authors concluded that this delay in appearance of oligoclonal bands after acute SARS-CoV-2 infections not initially involving neurologic symptoms could relate to development of a pathologic autoimmune response (58).

Prominent PASC complaints that are less quantifiable, including self-reported muscle pain, fatigue, and lethargy, may be amenable to objective study using electromyelograms, maximal electrically evoked twitch signals, and transcranial magnetic stimulation (87). Based on these investigations, it was hypothesized that PASC fatigue is a single entity with individual variation, rather than several distinct syndromes and pathophysiologies (87). Correlation with microthrombus and SARS-CoV-2 persistence in peripheral nerve tissues should be pursued. SARS-CoV-2 does infect the CNS, including those regions responsible for autonomic regulation, with defects leading to dysautonomia and postural orthostatic tachycardia syndrome, additional neurologic features of PASC, particularly among women (69).

### Cutaneous lesions and the microthrombus hypothesis for PASC.

Our group and others have defined two distinct types of cutaneous lesion in acute COVID-19: a thrombotic retiform purpura or livedo rash with endotheliopathy and microthrombosis on biopsy; and perniosis, occurring predominantly among those with only mild COVID-19, and not linked to microthrombosis (22, 88). An international registry of COVID-19 dermatologic findings found that lesions reported in PASC predominantly involved perniosis (89). However, there are also case reports of livedo, characterized by microthrombi, in PASC (89, 90). Punch biopsies of normal-appearing skin in individuals at the time of acute infection through development of PASC would be highly informative in terms of determining the significance of microthrombosis in the pathophysiology of PASC and divergent PASC phenotypes.

## EC pathology: PASC versus other severe respiratory infections

Studies involving PASC patients that include postbacterial ARDS and influenza controls are particularly informative. A total of 273,618 individuals were assessed 3 to 6 months after acute COVID-19 for nine common PASC symptoms: chest/throat pain, abnormal breathing, abdominal symptoms, fatigue, anxiety/depression, pain, headache, cognitive changes, and myalgia (91). All nine were more frequent after COVID-19 than after influenza, with hazard ratios of 1.44 to 2.04 (*P* < 0.001). This pattern was reflected by quantitative measurements in the United Kingdom Biobank study. A total of 384 controls, including those with a recent history of non–COVID-19 bacterial pneumonia or influenza, were compared with 386 non-hospitalized COVID-19 patients (92). Neurocognitive testing and structural and functional MRIs were performed at baseline and an average of 141 days after COVID-19 or other infection. Cognitive and imaging defects, including reduction in gray matter thickness and tissue contrast, were seen in the PASC patients versus both control groups (92).

Acute COVID-19 is also a trigger for AMI and stroke, as is influenza, but in the latter this risk is limited to a brief interval after infection (93, 94). During the first 3 days after influenza infection, the incidence ratio for AMI was 4.95, and that for stroke was 3.19 (91). By days 8–14 this risk declined markedly, to 1.71 and 1.51, respectively (94). In contrast, the incidence of AMI is five-fold greater than in hospitalized controls 14 days after COVID-19 diagnosis; that risk extends to 31 days after diagnosis, then declines (95). Similarly, the incidence of acute ischemic stroke is 10-fold higher than in hospitalized controls 14 days after COVID-19 diagnosis, extending to 31 days after diagnosis and then declining (95). In contrast to these phenomena, occurring within 31 days of acute COVID-19, AMI and stroke are infrequent in PASC. A large, self-controlled case series documented that while AMI and stroke have been observed in PASC (12), the greatest risk is in the first 2 to 4 weeks following acute SARS-CoV-2 infection (96). A similar national registry reported increased incidence rate ratios for DVT and pulmonary embolism extending to 70 days and 110 days, respectively, after acute COVID-19 (97). However, only pulmonary embolism risk would thus overlap with the lower limit for time interval in the WHO criteria for PASC. In addition, these complications were primarily restricted to PASC developing after critical acute COVID-19 (97). Occurrence of chronic kidney disease (CKD) also differs after COVID-19 ARDS versus bacterial ARDS. Intubated patients with COVID-19 pneumonia had a hazard ratio for developing PASC with CKD of 2.48 (*P* = 0.036) versus postbacterial ARDS, regardless of initial acute kidney injury stage (98).

The recent recognition of an association between incident DM and acute COVID-19 versus other respiratory infections and versus PASC is also of potential pathophysiologic importance. An increased risk of DM-1 holds for the first 30 days after SARS-CoV-2 infection, but not beyond (99). In contrast, DM-1 development is not a risk following pre–COVID-19 pandemic acute respiratory infections (100), and two studies of PASC developing after even mild acute COVID-19 found no risk for DM-1, but a hazard ratio of at least 1.40 for DM-2 (99, 101). Mechanisms are unclear, but this pattern did not correlate with preexisting risk factors for DM-2 or corticosteroid use (99, 101).

## SARS-CoV-2 reservoirs may underlie endotheliopathy in PASC

**Figure 2:**
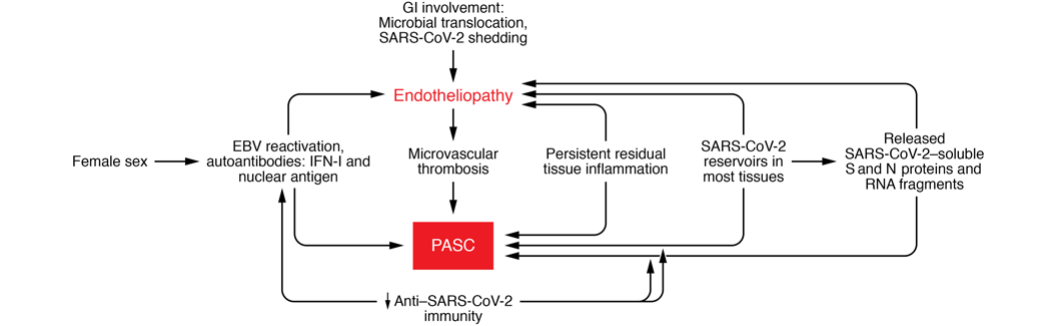
Potential mechanisms underlying long COVID (PASC). Multiple factors may contribute to the pathophysiology of at least certain PASC phenotypes, either directly or via microvascular injury. This occurs in the absence of the hypercoagulable state and systemic immune activation that characterize acute COVID-19. It is proposed that cryptic reservoirs of SARS-CoV-2 in multiple tissues, acting via direct EC infection, release of soluble viral products, infection of monocytes adjacent to the vasculature, or activation of other viruses, such as EBV, which can transmit virus to microvascular ECs, lead to cytopathic effects in ECs. Specific evidence for this is outlined in Tables 1 and 2. Autoantibodies, particularly against IFN-I, leading to suppression of the antiviral MxA pathway shown in Figure 1B, or against ECs, may also be involved. Development of SARS-CoV-2 cryptic reservoirs may be facilitated by lower antiviral immunity. Vaccinated individuals have a much lower incidence of PASC than the unvaccinated.

If systemic hyperinflammatory states do not subserve the endotheliopathy of long COVID, what might be the mechanism? Cryptic reservoirs of SARS-CoV-2 are strong candidates (Table 2). Extensive distribution of this virus in multiple tissues, including skin, heart, and brain, has been documented in autopsy series of acute COVID-19 patients. These data parallel studies demonstrating fibrin and platelet microthrombi in multiple organ systems at autopsy (20, 21, 24, 102). Although there is no clear relationship between PASC symptomatology and SARS-CoV-2 RNA levels in saliva at any time point after resolution of acute COVID-19 (71), prolonged viral fecal shedding is a key feature among many developing PASC, even in the absence of a positive nasopharyngeal test (2) (Figure 2). Similarly, SARS-CoV-2 RNA fragments have been found in 25% of PASC patients regardless of respiratory tract viral testing results (50). Anosmia is a common initial symptom of acute COVID-19 and, if it persists, a strong harbinger of PASC (103). This is important for two reasons. First, the severity of microvascular endotheliopathy seen in olfactory tissue in acute COVID-19 correlates with the extent of olfactory axonal damage (104). Second, even if non-neuronal cells are the main targets of the virus in the olfactory mucosa (105), these cells could be a source of persistent viral RNA in PASC. Murine coronavirus RNA persists in the CNS for prolonged periods in the absence of infectious virions and has been linked to demyelinating disorders (106).

Direct evidence for SARS-CoV-2 reservoirs in PASC derives from a comprehensive autopsy study of 44 patients conducted at the US NIH using droplet digital PCR to enable highly sensitive detection and quantification of SARS-CoV-2 in tissues (107). SARS-CoV-2 was widely distributed outside the respiratory tract more than 7 months after acute COVID-19 onset. This pattern was seen even among the few individuals who died of potentially unrelated issues with asymptomatic or only mild COVID-19 (107). The autopsy study supports an earlier autopsy-based study from Wuhan, China, of 26 patients examined by immunohistochemistry for SARS-CoV-2 spike (S) and nucleocapsid (N) proteins, which were detected at the endothelium in the lung and multiple extrapulmonary organs (108). It also parallels unique cases in which tissues from PASC patients with specific organ dysfunction have been obtained, as in the recent association of ED in PASC with endotheliopathy and SARS-CoV-2 expression in the penile microvasculature (79).

Establishment of a cryptic viral reservoir and persistent endotheliopathy may not require infection of ECs by SARS-CoV-2. The ability of SARS-CoV-2 to infect ECs is controversial (107, 109–111). But in vitro and murine models have shown induction of prothrombotic factors in microvascular ECs by isolated SARS-CoV-2 S and N proteins (16, 112–115), and other cell types adjacent to ECs, including monocytes and macrophages (108, 116), have detectable SARS-CoV-2 proteins (Figure 2). Albeit circulating SARS-CoV-2 RNA fragments are a prominent feature of PASC (50), such fragments, as well as soluble S and N proteins and viral pathogen-associated molecular patterns (PAMPs), could be epiphenomena rather than etiologic in EC injury. However, rodent models document the ability of SARS-CoV-2 S1 proteins to function as PAMPs, inducing localized CNS inflammation via pattern recognition receptor engagement and driving behavioral sickness responses in rats (16, 117). By analogy to subacute sclerosing panencephalitis, a very late neuropathology linked to measles virus infection caused by abnormal fusion proteins in the context of viral RNA, despite absence of infectious virions (106, 118), similar mechanisms may be involved in neurologic PASC.

SARS-CoV-2 persistence could also reactivate other latent viruses, such as Epstein-Barr virus (EBV), which may contribute to EC injury and PASC pathophysiology, or at least certain constitutional PASC symptoms (e.g., fatigue, myalgias) (119). EBV reactivation correlates with PASC (50). Although EBV cannot directly infect microvascular ECs, EBV-infected monocytes adjacent to ECs appear to be capable of transmitting EBV to those cells, with resultant cytopathic effects (120). Dermal microvascular injury and thrombosis linked to EBV have been described in scleroderma (120).

## PASC incidence and SARS-CoV-2 immune status

PASC is more common and severe in those with suboptimal anti–SARS-CoV-2 humoral and cellular immunity after acute infection. There is a lower frequency of CD8^+^ T cells expressing CD107a, a marker of degranulation, in response to SARS-CoV-2 peptides, and a more rapid decline in the frequency of SARS-CoV-2–specific IFN-γ–producing CD8^+^ T cells in those developing PASC (71). These immune defects could facilitate persistence of SARS-CoV-2 reservoirs, even if at very low levels. It is of particular concern for individuals with preexisting immunosuppressive disorders such as HIV (121). A recent study found that HIV status is strongly associated with PASC (odds ratio 4.01, *P* = 0.008) (122).

In terms of vaccination status, while peak SARS-CoV-2 viral loads are virtually identical following acute infection of vaccinated and unvaccinated individuals, those who are vaccinated have lower viral loads overall, and more rapidly clear virus (123). PASC risk among fully SARS-CoV-2–vaccinated individuals in cohorts from Israel, the United Kingdom, and the United States was markedly reduced in comparison with the unvaccinated (124–126). In one survey of 28,356 adults who received their first vaccination after SARS-CoV-2 infection, there was a 12.8% decline in PASC symptoms lasting more than 3 months, and a second vaccination correlated with an 8.8% decrease in PASC risk (125). The odds subsequently declined by 0.8% (range –1.2% to –0.4%) per week (125). An analysis of 25,225 SARS-CoV-2–vaccinated adults in the United States, from a cohort of 1,578,719 individuals with COVID-19, found significant differences in risks for hypertension and cardiac disease at 90 days between the vaccine and no-vaccine cohorts (126). If PASC were primarily driven by an inflammatory state, then immune activation by such vaccinations might have been expected to exacerbate such symptoms, not suppress them. It must be emphasized, however, that vaccination before SARS-CoV-2 infection confers only partial protection against PASC and thus “reliance on it as a sole mitigation strategy may not optimally reduce long-term health consequences of SARS-CoV-2 infection” (127).

### Autoantibody production.

Levels of autoantibodies linked to PASC susceptibility, described above, negatively correlate with anti–SARS-CoV-2 antibody concentrations (50). In that context, EC-activating autoantibodies, recognized in severe COVID-19 (128), could be involved in EC injury associated with PASC (Figure 2). This should be assessed. Exoproteome-targeting autoantibodies, which can perturb cell signaling and targeted killing of specific cell populations via Fc receptors and complement as well as suppress IL-1β, GM-CSF, and IL-21, are prominent in acute COVID-19 regardless of clinical stage (129). They can increase disease severity when administered to SARS-CoV-2–infected mice, but have not been explored in PASC. Whether any of these phenomena relate to a recently described “immunoglobulin signature” based on IgM and IgG3 measurements that, in combination with certain clinical symptoms during primary infection, predicts PASC development is unclear (130). Autoantibodies directed against IFN-I may be particularly important as they could impair IFN-I–initiated antiviral protein responses (Figure 1B). Our group documented suppression of the IFN-I–mediated antiviral protein MxA in the cutaneous microvasculature of critical versus mild and moderate acute COVID-19 patients (22, 88).

## *Quo vadis?* Conclusions and diagnostic and therapeutic implications

We agree with a recent review that “rigorous high dimensional profiling of tissues and peripheral blood, linking pathophysiological disruptions to clinical presentations and outcomes, will help delineate what are likely to be multiple syndromes that are still encapsulated in the term post-COVID-19 syndrome” (131). As summarized in Table 1, our focus on those clinical conditions amenable to investigation using quantitative methods suggests a link to endotheliopathy and microthrombosis, regardless of levels of systemic inflammation. Several alternative — in our view, complementary — mechanisms may also be involved in PASC (Figure 2). Table 2 summarizes evidence for persistent SARS-CoV-2 infection of cells related to the microvasculature that may underlie endothelial injury, even if the exact mechanisms — from direct EC infection to effects of soluble viral proteins on ECs to induction of specific autoantibodies and reactivation of other viruses that can affect ECs — are not yet established.

**Figure 3:**
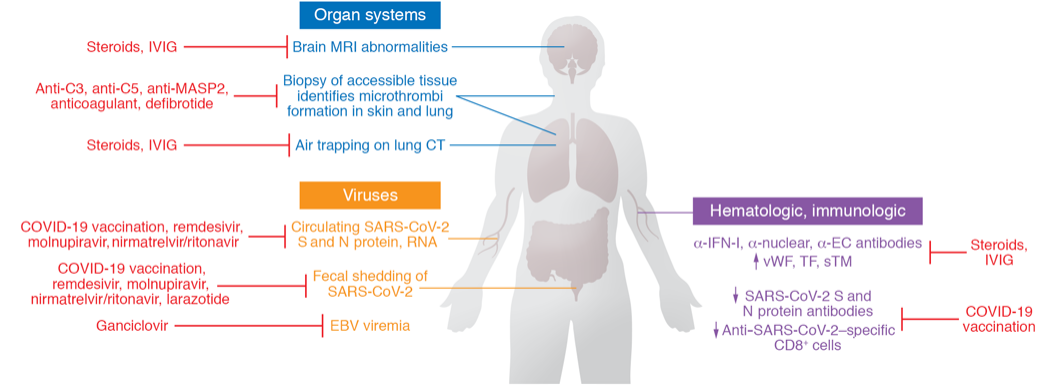
Proposed diagnostic assays and potential treatment targets in PASC. Abnormalities on lung CT and brain MRI have been correlated to functional changes, including dyspnea with air trapping and cognitive deficits, respectively, but they are not sensitive enough to distinguish an inflammatory versus a microthrombotic process. As PASC, like COVID-19, is a systemic process, we hypothesize that a simple 4 mm cutaneous punch biopsy of normal-appearing deltoid skin, a diagnostic method our group has employed for over a decade to investigate thrombotic microangiopathies linked to atypical hemolytic-uremic syndrome and hematopoietic stem cell transplantation and, most recently, acute COVID-19, should permit pathophysiologic explorations of PASC. Direct biopsy of other accessible tissues, including lung and peripheral nerve, could also be pursued. This could enable collection of evidence for vascular damage, microthrombi, and direct SARS-CoV-2 infection. Viral signals in stool and peripheral blood and hematologic/immunologic abnormalities linked to PASC may also be followed longitudinally. It should be recognized that the possible treatments illustrated are based on pathophysiology hypotheses and have not been evaluated in clinical trials. IVIG, intravenous immunoglobulin; sTM, soluble thrombomodulin.

Figure 3 summarizes potential candidates for diagnostic assays in PASC. All offer potential therapeutic targets, recognizing that these hypothesized interventions have not been evaluated in clinical trials. We postulate that a simple 4 mm cutaneous punch biopsy of normal-appearing deltoid skin, a diagnostic method our group has employed for over a decade to investigate thrombotic microangiopathies linked to atypical hemolytic-uremic syndrome and hematopoietic stem cell transplantation (132–134) and, most recently, acute COVID-19 (20, 22, 36, 88), should permit such pathophysiologic explorations of PASC. They could be accompanied by biopsy of other accessible involved organs or tissues, including the lung, GI tract, and peripheral nerve and, as reviewed here in the context of ED, the penis. This could enable collection of longitudinal evidence to support or refute endotheliopathy in the context of microthrombi and local SARS-CoV-2 reservoirs in PASC. Whether the complement deposition and tissue factor expression prominent in skin and lung of critical COVID-19 patients (20, 22, 102) also characterize PASC could also be determined.

If the role we propose for EC injury in PASC, whether central or simply an important contributory factor, parallels its dominance in the pathology of acute COVID-19, at least some of the interventions utilized in the latter, including dexamethasone, baricitinib, anticomplement (C3, C5, MASP-2) agents, and defibrotide (20, 22, 37, 38, 61, 135, 136), might be evaluated in rodent models for acute SARS-CoV-2 infection (106, 137), then tested clinically (Figure 3). Certain of these models develop acute systemic microthrombosis (106). Anticoagulation in acute COVID-19 correlates with a 4-day reduction in SARS-CoV-2 positivity on nasopharyngeal sampling and a reduction in mortality, despite an absence of effect on development of markers for COVID-19–associated coagulopathy (29). Its utility in PASC is unknown. Given the absence of fibrinolytic activity in heparins, our group explored alternative anticoagulants such as argatroban, which does have such properties, in severe acute COVID-19 (138). As plasmin-resistant microclots in association with complement have been described in PASC (80), this may be another fruitful area to explore. GI breach of SARS-CoV-2 viral particles from the gut lumen into the systemic circulation has been linked to the multisystem inflammatory syndrome (MIS) of both acute COVID-19 and PASC in children (32). This breach is facilitated by zonulin, which loosens tight junctions between epithelial cells, and is mitigated by the zonulin inhibitor larazotide (33). Addition of larazotide to the immunosuppressive regimens used in MIS in a pediatric cohort led to significantly improved time to resolution of GI symptoms and time to clearance of SARS-CoV-2 S protein antigenemia (33) and might be evaluated in PASC in adults.

Finally, SARS-CoV-2 antivirals, including the FDA-approved remdesivir, molnupiravir, and nirmatrelvir/ritonavir, and drugs that synergize with those nucleoside analogs (139, 140), could be tested. If EBV reactivation is confirmed as a significant contributor to at least some manifestations of PASC, anti-herpesvirus agents may have some promise. Although there are currently no agents approved to specifically treat EBV reactivation (119), ganciclovir, which inhibits replication of EBV and cytomegalovirus, has, in an observational study, reduced the risk of death in patients with severe COVID-19 (141). The need for evaluation of potential interventions is of growing concern as new data suggest that the risk of severe outcomes in acute COVID-19, and the risk for development of long COVID, increases in a graded fashion according to the number of SARS-CoV-2 infections experienced, regardless of vaccination status (142).

## Figures and Tables

**Table 1:**
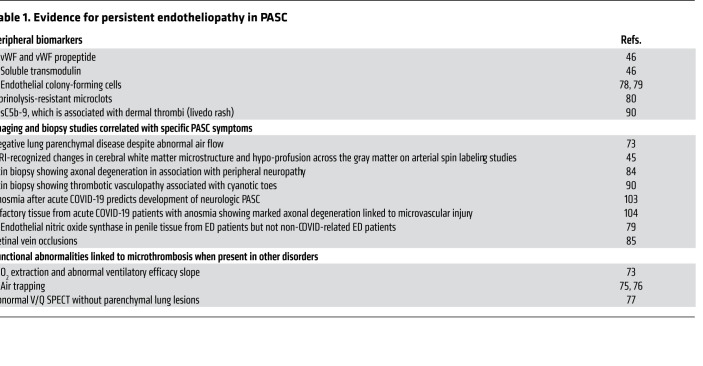
Evidence for persistent endotheliopathy in PASC

**Table 2:**
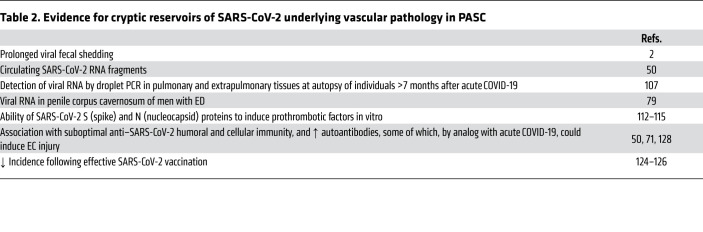
Evidence for cryptic reservoirs of SARS-CoV-2 underlying vascular pathology in PASC

## Version 1 08/01/2022

Electronic publication
